# The impact of hospital saturation on non-COVID-19 hospital mortality during the pandemic in France: a national population-based cohort study

**DOI:** 10.1186/s12889-024-19282-3

**Published:** 2024-07-05

**Authors:** Laurent Boyer, Vanessa Pauly, Yann Brousse, Veronica Orleans, Bach Tran, Dong Keon Yon, Pascal Auquier, Guillaume Fond, Antoine Duclos

**Affiliations:** 1 CEReSS - Health Service Research and Quality of Life Center, UR3279, Aix-Marseille University, APHM, Marseille, 13005 France; 2 Institute of Preventive Medicine and Public Health, Hanoi Medical University, Hanoi, 100000 Vietnam; 3 Center for Digital Health, Medical Science Research Institute, Kyung Hee University College of Medicine, Seoul, South Korea; 4 Department of Pediatrics, Kyung Hee University Medical Center, Kyung Hee University College of Medicine, Seoul, South Korea; 5 RESHAPE - Research on Healthcare Performance Lab, Inserm U1290, Claude Bernard Lyon 1 University, Lyon, 69424 France

**Keywords:** Health services research, Public health, COVID-19

## Abstract

**Background:**

A previous study reported significant excess mortality among non-COVID-19 patients due to disrupted surgical care caused by resource prioritization for COVID-19 cases in France. The primary objective was to investigate if a similar impact occurred for medical conditions and determine the effect of hospital saturation on non-COVID-19 hospital mortality during the first year of the pandemic in France.

**Methods:**

We conducted a nationwide population-based cohort study including all adult patients hospitalized for non-COVID-19 acute medical conditions in France between March 1, 2020 and 31 May, 2020 (1st wave) and September 1, 2020 and December 31, 2020 (2nd wave). Hospital saturation was categorized into four levels based on weekly bed occupancy for COVID-19: no saturation (< 5%), low saturation (> 5% and ≤ 15%), moderate saturation (> 15% and ≤ 30%), and high saturation (> 30%). Multivariate generalized linear model analyzed the association between hospital saturation and mortality with adjustment for age, sex, COVID-19 wave, Charlson Comorbidity Index, case-mix, source of hospital admission, ICU admission, category of hospital and region of residence.

**Results:**

A total of 2,264,871 adult patients were hospitalized for acute medical conditions. In the multivariate analysis, the hospital mortality was significantly higher in low saturated hospitals (adjusted Odds Ratio/aOR = 1.05, 95% CI [1.34–1.07], *P* < .001), moderate saturated hospitals (aOR = 1.12, 95% CI [1.09–1.14], *P* < .001), and highly saturated hospitals (aOR = 1.25, 95% CI [1.21–1.30], *P* < .001) compared to non-saturated hospitals. The proportion of deaths outside ICU was higher in highly saturated hospitals (87%) compared to non-, low- or moderate saturated hospitals (81–84%). The negative impact of hospital saturation on mortality was more pronounced in patients older than 65 years, those with fewer comorbidities (Charlson 1–2 and 3 vs. 0), patients with cancer, nervous and mental diseases, those admitted from home or through the emergency room (compared to transfers from other hospital wards), and those not admitted to the intensive care unit.

**Conclusions:**

Our study reveals a noteworthy “dose-effect” relationship: as hospital saturation intensifies, the non-COVID-19 hospital mortality risk also increases. These results raise concerns regarding hospitals’ resilience and patient safety, underscoring the importance of identifying targeted strategies to enhance resilience for the future, particularly for high-risk patients.

**Supplementary Information:**

The online version contains supplementary material available at 10.1186/s12889-024-19282-3.

## Background

Over the past two years, the surge in patients with SARS-CoV-2 coronavirus (COVID-19) has placed a significant strain on hospital capacities, exhausting healthcare resources such as staff, beds, and equipment [1, 2]. France, like many other countries, experienced two substantial waves of COVID-19 cases from March 2020 to December 2020, leading to nationwide lockdowns and the cancellation of planned medical care [3]. The excessive workload and resource allocation toward COVID-19 cases may have disrupted non-COVID-19 care and had an impact on health outcomes [4].

A previous study highlighted the disruption of surgical care for non-COVID-19 patients and reported a significant increase in excess mortality in France [5]. This study revealed a notable rise in mortality among non-COVID-19 surgical patients in hospitals simultaneously managing COVID-19 cases in their intensive care units (ICU). The excess mortality primarily occurred outside of the ICU [5]. Other studies conducted in France have also documented deteriorating access, effectiveness, and safety in non-COVID-19 primary care [6], perinatal care [7] and abortion [8], and acute care in vulnerable patients such as those with severe mental disorders [9]. Similar trends have been observed in other countries. Access to care issues have been reported across various studies focusing on conditions such as acute physical and mental conditions [10], severe asthma [11], and cardiovascular diseases [12, 13]. Similarly, issues related to effectiveness and safety have been identified for patients with diabetes [14], ICU patients [15, 16], and long-term care facility residents [17]. Additionally, studies have found higher maternal mortality rates [18]. However, the extent to which the excessive workload and resource prioritization toward COVID-19 care have affected safety of non-COVID-19 acute medical care remains unknown.

The primary objective of this study was to assess the impact of hospital beds saturation on non-COVID-19 hospital mortality for acute medical conditions during the first year of the pandemic in France. Building on previous findings in the surgical setting, we hypothesized that high hospital bed occupancy due to COVID-19 cases may have compromised medical safety [5]. The secondary objective was to compare the clinical profile of non-COVID-19 patients based on hospital saturation rates.

## Methods

### Study design, sources and population

In this nationwide population-based cohort study, we used data from the Programme de Médicalisation des Systèmes d’Information (PMSI database), the French national hospital database in which administrative and medical data are systematically collected for acute (PMSI-MCO) care. The healthcare landscape in France encompasses a total of 3,008 establishments: 1,354 of these are public hospitals, while 1,654 are private hospitals [19]. These facilities span a wide range of medical disciplines, including medicine, surgery, obstetrics (both acute and ambulatory care), psychiatry, post-acute care, rehabilitation, and home hospitalization. Our study specifically focuses on hospitals that offer medical acute care services. The PMSI database is based on diagnosis-related groups with all diagnoses coded according to the 10th version of the International Classification of Diseases (ICD-10) and procedural codes from the Classification Commune des Actes Médicaux (CCAM). The coding process involves a combination of manual and artificial intelligence coding techniques, ensuring comprehensive and accurate data representation [20]. The PMSI database is used to determine financial resources and is frequently and thoroughly verified by both its producer and the paying party with possible financial and legal consequences [21]. The manuscript follows the REporting of studies Conducted using Observational Routinely-collected health Data (RECORD) Statement [22]. We included all public and private hospital admissions between March 1, 2020 and 31 May, 2020 (1st wave) and September 1, 2020 and December 31, 2020 (2nd wave) according to the following criteria: aged 18 years or older, admitted for acute care without COVID-19 (ICD-10 codes different from U071, U0710, U0711, U0712, U0714, U0715 in PMSI-MCO, these ICD-10 codes have been reported to be valid for detecting COVID-19 hospital stays [23]), for medical condition (exclusion of stay with surgery or obstetrical stays, defined by the presence of at least one procedural code related to surgery, obstetrics, or maternity care) and a length of hospital stay > 24 h (to exclude ambulatory care) except if the patients died within 24 h. Were excluded stays from hospitals with no available data concerning the number of conventional beds, transferred patients within 48 h and stays in non-metropolitan areas (overseas departments and territories which are located outside the European continent)because of substantial differences in healthcare infrastructure, resources, and access to specialized care from the mainland [24].

### Procedure

We defined four groups according to the quartile of the saturation rate (number of patients hospitalized for COVID-19/number of hospital beds) [25]: absence (< 5%) / low ([5–15%[) / moderate ([15–30%[) / high (> 30%) saturation (Supplementary Fig. 1). The calculation was performed for each calendar week during each wave. The hospital saturation rate at the time of admission was considered for each stay. The unit of analysis was the stay, not the patient.

### Outcomes

The primary outcome was hospital mortality from all causes (index stay). The secondary outcome was the patient case-mix based on ICD-10 chapters (i.e., admission-diagnosis distribution).

### Collected data

In addition to mortality and patient case-mix, we collected the following data: age, sex, comorbidities using the Charlson Comorbidity Index (0, 1 to 2, ≥ 3 [26]), characteristics of the stay including the source of hospital admission (i.e., where the patient came from [home, emergency, transfer from other hospital ward]), ICU admission, and durations of ICU and hospital stay. Characteristics of the hospital (academic, general public, and private) and region of residence were also recorded.

### Statistical analysis

The patients’ characteristics are presented as counts (percentages) and medians (interquartile ranges) for categorical and continuous variables, respectively. To study the association between saturation and mortality, univariate and multivariate generalized linear models (i.e., logistic model with hospital as a random effect to take into cluster effect due to correlation of data within hospitals) were used, odds ratio (ORs) and 95% confidence intervals (95% CIs) were estimated.

In addition to the saturation rate, the multivariate model incorporated the following variables (with *P* < .05 in the univariate analysis): age classes, sex, COVID-19 wave, Charlson Comorbidity Index, case-mix, source of hospital admission, ICU admission, category of hospital and region of residence. Multicollinearity was evaluated using the Variance Inflation Factor (VIF) for each independent variable, complemented by analyses involving sequential exclusion of one variable at a time. The quality of the model was assessed using the deviance statistic and the area under the curve (AUC).

Interaction terms between saturation and several factors (age, sex, ICD-10 chapters, Charlson comorbidities, source of hospital admission, ICU admission and characteristics of the hospital) were tested to determine whether the association between saturation and mortality was homogeneous and to identify at-risk subgroups.

Three sensitivity analyses were conducted to assess the robustness of our primary multivariate analysis. In the first analysis, we substituted the Charlson Comorbidity Index with individual comorbidities. In the second analysis, the saturation rate categories were replaced with the numeric continuous variable. In the third analysis, we utilized a Cox proportional hazards regression analysis with a shared frailty factor (representing the hospital) instead of the multivariate generalized linear models. The endpoint for this analysis was defined as the time to death (event) or discharge alive from the hospital (censoring). We conducted this third sensitivity analysis to assess whether the risk factors, especially hospital saturation, have an impact on the time to death, in contrast to generalized linear models that only evaluate death as a binary event.

A *P* < .05 was considered significant. Data management and analyses were performed using the SAS software ^®^ including the PROC GLIMMIX for generalized linear models and PROC PHREG with random statement for shared frailty models.

### Ethical approval and consent to participate

Data from the PMSI database are anonymized and can be reused for research purposes as done in previous works [9, 27–29]. In accordance with the French law [30] and the General Data Protection Regulation (GDPR), informed consent from its participants was waived and the study was approved by the French National Data Protection Commission (No. F20211214152715 [31]).

## Results

**Fig. 1:**
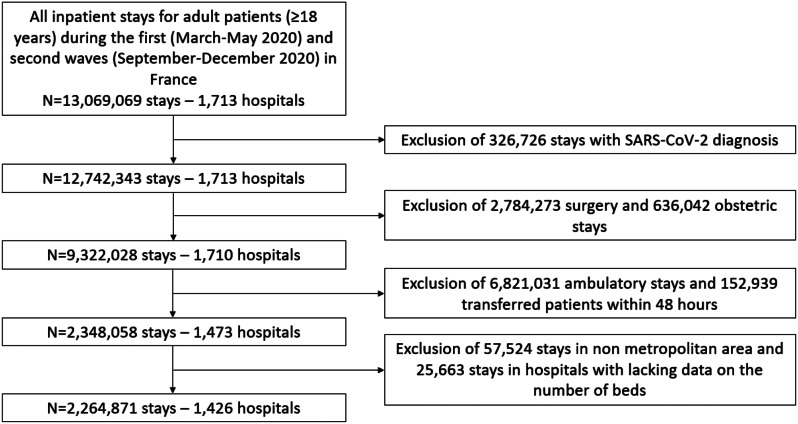
Flowchart

The database consisted of a total of 2,264,871 stays admitted to 1,426 hospitals for non-COVID-19 acute medical conditions (flow chart, Fig. 1). Tables 1 and 2 provide an overview of the baseline characteristics of the entire patient sample categorized by hospital saturation levels and their status (alive or deceased). The mean age was 66.59 (± 26) years, and 53.4% were male. The majority of patients (1,855,574 patients [81.9%]) were hospitalized in public hospitals. Among the hospital stays included in this study, 85,300 stays (4%) occurred in highly saturated hospitals, 281,701 stays (12%) in moderately saturated hospitals, 685,110 stays (30%) in low saturated hospitals, and 1,212,760 stays (54%) in non-saturated hospitals.

**Table 1:** Characteristics of patients according to hospital saturation level

Characteristics	No saturated hospitals *N* = 1,212,760 (53.5%)	Low saturated hospitals *N* = 685,110 (30.2%)	Moderate saturated hospitals *N* = 281,701 (12.5%)	Highly saturated hospitals *N* = 85,300 (3.8%)	Absolute Difference (%)*	P**	P***
**Socio-demographic characteristics**							
**Age (mean ± std)**	66.93 ± 18.03	66.11 ± 18.70	66.11 ± 18.89	67.08 ± 19.19		**< 0.001**	**< 0.001**
**Age classes, year**						**< 0.001**	**< 0.001**
18–24	29,243 (2.4%)	20,010 (2.9%)	8,350 (3.0%)	2,341 (2.7%)	0.3%		
25–34	51,077 (4.2%)	33,822 (4.9%)	14,249 (5.1%)	4,331 (5.1%)	0.9%		
35–44	73,630 (6.1%)	45,509 (6.6%)	19,130 (6.8%)	5,842 (6.9%)	0.8%		
45–54	125,755 (10.4%)	73,010 (10.7%)	30,440 (10.8%)	8,655 (10.2%)	-0.2%		
55–64	192,507 (15.9%)	107,288 (15.7%)	43,304 (15.4%)	12,249 (14.4%)	-1.5%		
65–74	276,604 (22.8%)	148,456 (21.7%)	59,056 (21.0%)	16,648 (19.5%)	-3.3%		
75–84	250,146 (20.6%)	136,097 (19.9%)	56,111 (19.9%)	17,351 (20.3%)	-0.3%		
85–94	191,406 (15.8%)	108,234 (15.8%)	45,566 (16.2%)	15,782 (18.5%)	2.7%		
≥ 95	22,392 (1.9%)	12,684 (1.9%)	5,495 (2.0%)	2,101 (2.5%)	0.6%		
**Sex**						**< 0.001**	**< 0.001**
Male	649,843 (53.6%)	367,042 (53.6%)	149,692 (53.1%)	44,551 (52.2%)	-1.4%		
**Comorbidities**							
**Charlson score**						**< 0.001**	**< 0.001**
0	415,674 (34.3%)	228,903 (33.4%)	91,807 (32.6%)	27,499 (32.2%)	-2.1%		
1–2	388,415 (33.7%)	224,618 (32.8%)	98,887 (35.1%)	27,284 (32.0%)	-1.7%		
≥ 3	408,671 (33.7%)	231,589 (33.8%)	98,887 (35.1%)	30,517 (35.8%)	2.1%		
**Charlson comorbidities**							
Renal disease	126,000 (10.4%)	77,533 (11.3%)	33,350 (11,8%)	11,070 (13.0%)	2.6%	**< 0.001**	**< 0.001**
Liver mild disease	54,497 (4.5%)	35,040 (5.1%)	14,961 (5.3%)	4,378 (5.1%)	0.6%	**< 0.001**	**< 0.001**
Liver moderate / severe disease	24,437 (2.0%)	15,399 (2.3%)	6,643 (2.4%)	1,893 (2.2%)	0.2%	**< 0.001**	**< 0.001**
Peptic ulcer	17,429 (1.4%)	10,386 (1.5%)	4,400 (1.6%)	1,299 (1.5%)	0.1%	**< 0.001**	**< 0.001**
Chronic pulmonary disease	129,354 (10.7%)	77,679 (11.3%)	33,989 (12.1%)	11,114 (13.0%)	2.3%	**< 0.001**	**< 0.001**
Congestive heart failure	223,652 (18.4%)	134,354 (19.6%)	56,567 (20.1%)	17,726 (20.8%)	2.4%	**< 0.001**	**< 0.001**
Myocardial infarction	130,250 (10.7%)	78,015 (11.4%)	31,403 (11.2%)	9,063 (10.6%)	-0.1%	**< 0.001**	**< 0.001**
Peripheral vascular disease	104,907 (8.7%)	57,558 (8.4%)	23,739 (8.4%)	6,872 (8.1%)	-0.6%	**< 0.001**	**< 0.001**
Cerebrovascular disease	111,093 (9.2%)	73,776 (10.8%)	31,085 (11.0%)	9,324 (10.9%)	1.7%	**< 0.001**	**< 0.001**
Dementia	71,240 (5.9%)	43,893 (6.4%)	19,782 (7.0%)	7,823 (9.2%)	3.3%	**< 0.001**	**< 0.001**
Hemi/Paraplegia	60,020 (5.0%)	38,895 (5.7%)	16,582 (5.9%)	4,7343 (5.6%)	0.6%	**< 0.001**	**< 0.001**
Rheumatic disease	19,671 (1.6%)	12,739 (1.9%)	4,909 (1.7%)	1,331 (1.6%)	0.0%	**< 0.001**	**< 0.001**
Metastatic solid tumor	131,669 (10.9%)	58,973 (8.6%)	25,282 (9.0%)	7,982 (9.4%)	-1.5%	**< 0.001**	**< 0.001**
Malignancy	270,270 (22.3%)	132,187 (19.3%)	55,235 (19.6%)	16,516 (19.4%)	-2.9%	**< 0.001**	**< 0.001**
Non-Complicated diabetes	201,757 (16.6%)	120,939 (17.7%)	51,904 (18.4%)	15,561 (18.2%)	1.6%	**< 0.001**	**< 0.001**
Complicated diabetes	62,961 (5.2%)	43,095 (6.3%)	18,934 (6.7%)	5,629 (6.6%)	1.4%	**< 0.001**	**< 0.001**
AIDS/HIV	3,838 (0.3%)	3,245 (0.5%)	1,410 (0.5%)	419 (0.5%)	0.2%	**< 0.001**	**< 0.001**
**Characteristics of stay**							
**Origin of the patient**						**< 0.001**	**< 0.001**
Home	599,992 (49.5%)	283,395 (41.4%)	105,653 (37.5%)	267,502 (31.3%)	-18.2%		
Emergency ward	493,886 (40.7%)	325,529 (51.5%)	153,981 (54.7%)	49,160 (57.6%)	16.9%		
Transfer from other hospital	118,882 (9.8%)	49,186 (7.2%)	22,067 (7.8%)	9,438 (11.1%)	1.3%		
**Length of stay (mean ± sd)**	5.99 ± 7.84	6.12 ± 7.79	6.23 ± 6.71	6.09 ± 7.81		**< 0.001**	**< 0.001**
**ICU care**							
ICU admission	156,950 (12.9%)	98,566 (14.4%)	38,011 (13.5%)	10,047 (11.8%)	-1.1%	**< 0.001**	**< 0.001**
Delay of admission to ICU in 1 day	128,468 (81.9%)	81,995 (83.2%)	31,973 (84.1%)	8,549 (85.1%)	3.2%	**< 0.001**	**< 0.001**
**Length of ICU stay (mean ± sd)**	4.38 ± 6.16	4.34 ± 6.33	4.28 ± 6.2	4.35 ± 6.23		**< 0.001**	**< 0.001**
**Casemix based on ICD-10 chapters**						**< 0.001**	**< 0.001**
Infectious diseases	26,077 (2.2%)	16,663 (2.4%)	7,022 (2.5%)	2,388 (2.8%)	0.6%		
Cancer	157,924 (13.0%)	69,720 (10.2%)	29,105 (10.3%)	8,369 (9.8%)	-3.2%		
Haematological disorders	40,092 (3.3%)	22,454 (3.3%)	9,698 (3.4%)	3,149 (3.7%)	0.4%		
Endocrine, nutrition, and metabolism	46,854 (3.9%)	28,898 (4.2%)	11,891 (4.2%)	3,327 (3.9%)	0.0%		
Diseases of the nervous system	67,547 (5.6%)	39,054 (5.8%)	15,308 (5.4%)	3,997 (4.7%)	-0.9%		
Sensory organ disease	9,481 (0.8%)	6,712 (1.0%)	2,534 (0.9%)	657 (0.8%)	0.0%		
Circulatory disease	276,312 (22.8%)	159,665 (23.3%)	62,563 (22.2%)	16,993 (19.9%)	-2.9%		
Respiratory disease	86,734 (7.2%)	55,081 (8.0%)	24,372 (8.7%)	9,262 (10.9%)	3.7%		
Digestive disease	103,265 (8.5%)	56,130 (8.2%)	22,642 (8.0%)	6,640 (7.8%)	-0.7%		
Dermatological disease	12,950 (1.1%)	7,943 (1.2%)	3,295 (1.2%)	856 (1.0%)	-0.1%		
Bone, muscle and connective tissues	40,928 (3.4%)	23,852 (3.5%)	9,098 (3.2%)	2,563 (3.0%)	-0.4%		
Genitourinary system	62,147 (5.1%)	35,757 (5.2%)	14,945 (5.3%)	4,847 (5.7%)	0.6%		
Injury and poisoning	43,390 (3.6%)	28,079 (4.1%)	12,227 (4.3%)	3,908 (4.6%)	1.0%		
Mental disorders	55,817 (4.6%)	35,884 (5.2%)	15,810 (5.6%)	5,454 (6.4%)	1.8%		
Others	183,242 (15.1%)	98,768 (14.4%)	41,191 (14.6%)	12,890 (15.1%)	0.0%		
**Hospital characteristics**						**< 0.001**	**< 0.001**
Academic	313,122 (25.8%)	218,481 (31.9%)	83,329 (29.6%)	19,109 (22.4%)	-3.4%		
Other public hospital	576,371 (47.5%)	402,986 (58.8%)	181,824 (64.5%)	60,352 (70.8%)	23.3%		
Private	323,267 (26.7%)	63,643 (9.3%)	16,548 (5.9%)	5,839 (6.9%)	-19.8%		

* Absolute difference between highly and non-saturated hospitals; ** Statistical significance between the four groups; *** Statistical significance for trend test

Nationwide population-based cohort study; All adult patients hospitalized for non-COVID-19 acute medical conditions; France; Between March 1, 2020 and 31 May, 2020 (1st wave) and September 1, 2020 and December 31, 2020 (2nd wave)

**Table 2:** Characteristics of patients, univariate and multivariate analysis

	Whole sample *N* = 2,264,871 (100.0%)	Alive *N* = 2,140,481 (94.5%)	Dead *N* = 124,390 (5.5%)	P*	Adjusted OR	P**
**Saturation**				**< 0.001**		
< 5% (no)	1,212,760 (53.6%)	1,148,504 (53.7%)	64,256 (51.7%)		1	---
[5–15%[ (low)	685,110 (30.2%)	647,696 (30.3%)	37,414 (30.1%)		1.05 [1.34;1.07]	**< 0.001**
[15–30%[ (moderate)	281,701 (12.4%)	264,966 (12.4%)	16,735 (13.5%)		1.12 [1.09;1.14]	**< 0.001**
> 30% (high)	85,300 (3.8%)	79,315 (3.7%)	5,985 (4.8%)		1.25 [1.21;1.29]	**< 0.001**
**Age classes, year**				**< 0.001**		
18–24	59,944 (2.7%)	59,735 (2.8%)	209 (0.2%)		1	---
25–34	103,479 (4.6%)	102,908 (4.8%)	571 (0.5%)		1.50 [1.28;1.77]	**< 0.001**
35–44	144,111 (6.4%)	142,409 (6.7%)	1,702 (1.4%)		2.62 [2.26;3.03]	**< 0.001**
45–54	237,860 (10.5%)	232,476 (10.9%)	5,384 (4.3%)		3.79 [3.21;4.36]	**< 0.001**
55–64	355,348 (15.7%)	341,464 (16.0%)	13,884 (11.2%)		5.13 [4.46;5.89]	**< 0.001**
65–74	500,764 (22.1%)	474,665 (22.2%)	26,099 (21.0%)		6.34 [5.52;7.28]	**< 0.001**
75–84	459,705 (20.3%)	427,918 (20.0%)	31,787 (25.6%)		8.91 [7.76;10.23]	**< 0.001**
85–94	360,988 (15.9%)	322,808 (15.1%)	38,180 (30.7%)		15.64 [13.63;17.96]	**< 0.001**
≥ 95	42,672 (1.9%)	36,098 (1.7%)	6,574 (5.3%)		28.17 [24.48;32.42]	**< 0.001**
**Sex**				**< 0.001**		
Female	1,053,743 (46.5%)	997,661 (46.6%)	56,082 (45.1%)		1	---
Male	1,211,128 (53.5%)	1,142,820 (53.4%)	68,308 (54.9%)		1.07 [1.05;1.08]	**< 0.001**
**COVID-19 wave**				**< 0.001**		
1st wave	886,134 (39.1%)	835,417 (39.0%)	50,717 (40.8%)		1	---
2nd wave	1,378,737 (60.9%)	1,305,064 (61.0%)	73,673 (59.2%)		1.05 [1.04;1.07]	**< 0.001**
**Charlson comorbidity score**				**< 0.001**		**< 0.001**
0	763,883 (33.7%)	752,533 (35.2%)	11,350 (9.1%)		1	---
1–2	731,324 (32.3%)	699,416 (32.7%)	31,908 (25.7%)		1.57 [1.53;1.61]	**< 0.001**
≥ 3	769,664 (34.0%)	688,532 (32.2%)	81,132 (65.2%)		3.20 [3.13;3.27]	**< 0.001**
**Case-mix**				**< 0.001**		
Others	336,091 (14.8%)	324,650 (15.2%)	11,441 (9.2%)		1	---
Infectious diseases	52,150 (2.3%)	49,306 (2.3%)	2,844 (2.3%)		1.23 [1.18;1.28]	**< 0.001**
Cancer	265,118 (11.7%)	225,789 (10.6%)	39,329 (31.6%)		5.20 [5.08;5.33]	**< 0.001**
Haematological disorders	75,393 (3.3%)	73,705 (3.4%)	1,688 (1.4%)		0.46 [0.44;0.49]	**< 0.001**
Endocrine, nutrition, and metabolism	90,970 (4.0%)	88,482 (4.1%)	2,488 (2.0%)		0.75 [0.72;0.79]	**< 0.001**
Diseases of the nervous system	126,356 (5.6%)	123,734 (5.8%)	2,622 (2.1%)		0.65 [0.62;0.68]	**< 0.001**
Sensory organ disease	19,384 (0.9%)	19,362 (0.9%)	22 (0.0%)		0.03 [0.02;0.05]	**< 0.001**
Circulatory disease	515,533 (22.8%)	486,432 (22.7%)	29,101 (23.4%)		0.96 [0.93;0.98]	0.001
Respiratory disease	175,449 (7.8%)	156,082 (7.3%)	19,367 (15.6%)		2.19 [2.13;2.24]	**< 0.001**
Digestive disease	188,677 (8.3%)	182,309 (8.5%)	6,368 (5.1%)		0.99 [0.96;1.02]	0.63
Dermatological disease	25,044 (1.1%)	24,343 (1.1%)	701 (0.6%)		0.78 [0.72;0.85]	**< 0.001**
Bone, muscle and connective tissues	76,441 (3.4%)	75,761 (3.5%)	680 (0.6%)		0.28 [0.25;0.30]	**< 0.001**
Genitourinary system	117,696 (5.2%)	113,629 (5.3%)	4,067 (3.3%)		0.76 [0.73;0.78]	**< 0.001**
Injury and poisoning	87,604 (3.9%)	85,064 (4.0%)	2,540 (2.0%)		0.81 [0.77;0.85]	**< 0.001**
Mental disorders	112,965 (5.0%)	111,833 (5.2%)	1,132 (0.9%)		0.33 [0.31;0.35]	**< 0.001**
**Source of hospital admission**				**< 0.001**		
Home	1,015,742 (44.9%)	981,170 (45.8%)	34,572 (27.8%)		1	---
Emergency	1,049,556 (46.3%)	982,075 (45.9%)	67,481 (54.3%)		2.36 [2.32 ;2.40]	**< 0.001**
Transfer from other hospital ward	199,573 (8.8%)	177,236 (8.3%)	22,337 (18.0%)		2.38 [2.33 ;2.43]	**< 0.001**
**ICU admission**				**< 0.001**		
No	1,961,297 (86.6%)	1,865,203 (87.1%)	96,094 (77.3%)		1	---
Yes	303,574 (13.4%)	275,278 (12.9%)	28,296 (22.8%)		2.73 [2.68;2.77]	**< 0.001**
**Category of hospital**				**< 0.001**		
Other public hospital	1,221,533 (53.9%)	1,141,323 (53.3%)	80,210 (64.5%)		1	---
Academic	634,041 (28.0%)	604,849 (28.3%)	29,192 (23.5%)		0.65 [0.56;0.75]	**< 0.001**
Private	409,297 (18.1%)	394,309 (18.4%)	14,988 (12.1%)		0.42 [0.38;0.47]	**< 0.001**

*Univariate analysis; **Multivariate analysis

Nationwide population-based cohort study; All adult patients hospitalized for non-COVID-19 acute medical conditions; France; Between March 1, 2020 and 31 May, 2020 (1st wave) and September 1, 2020 and December 31, 2020 (2nd wave)

## Hospital mortality

**Fig. 2:**
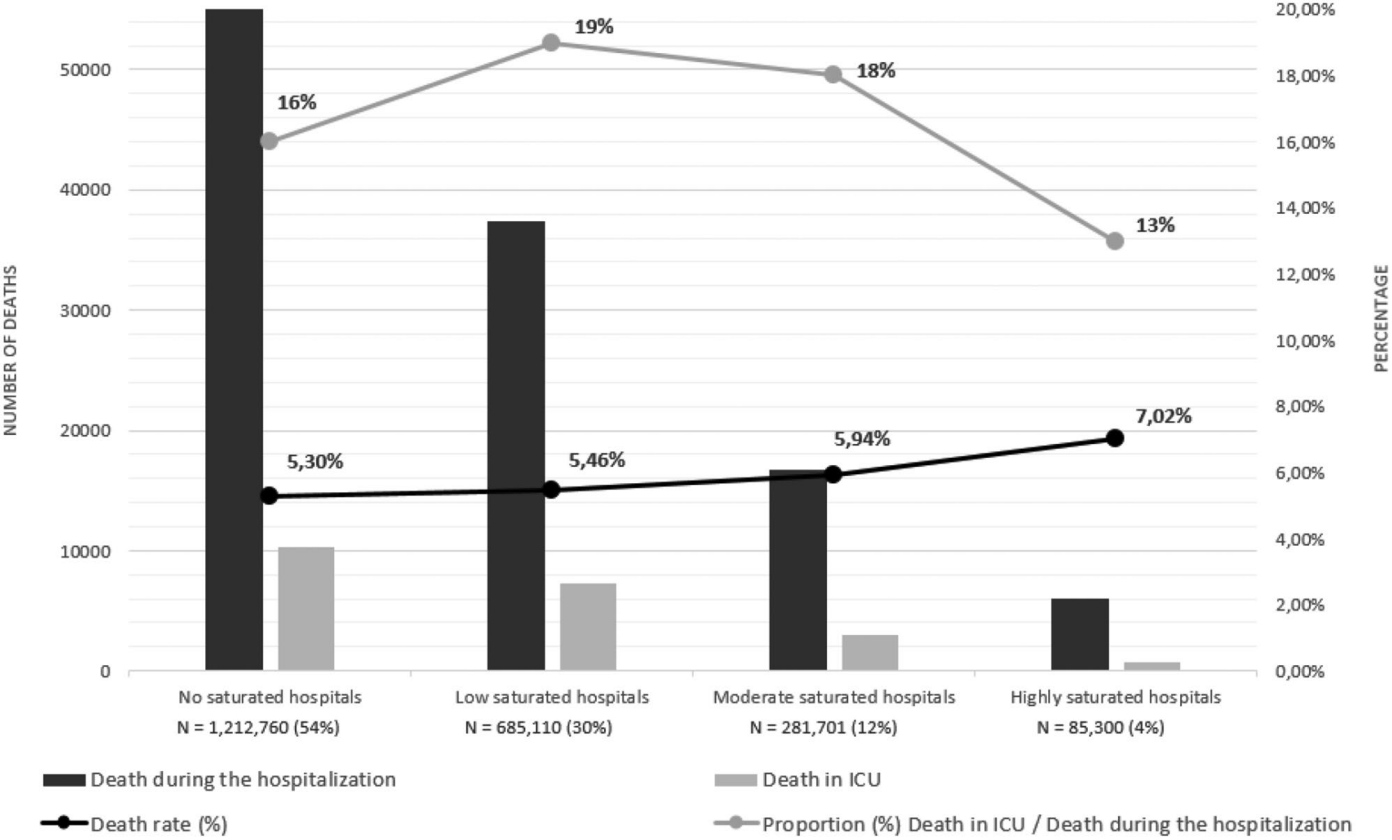
Mortality according to the hospital saturation. The proportion of deaths within the ICU was lower in highly saturated hospitals (13%) compared to non- (16%), low- (19%), or moderate saturated (18%) hospitals. Conversely, the proportion of deaths occurring outside the ICU was higher in highly saturated hospitals (87%) compared to non- (84%), low- (81%), or moderate saturated (82%) hospitals

**Fig. 3:**
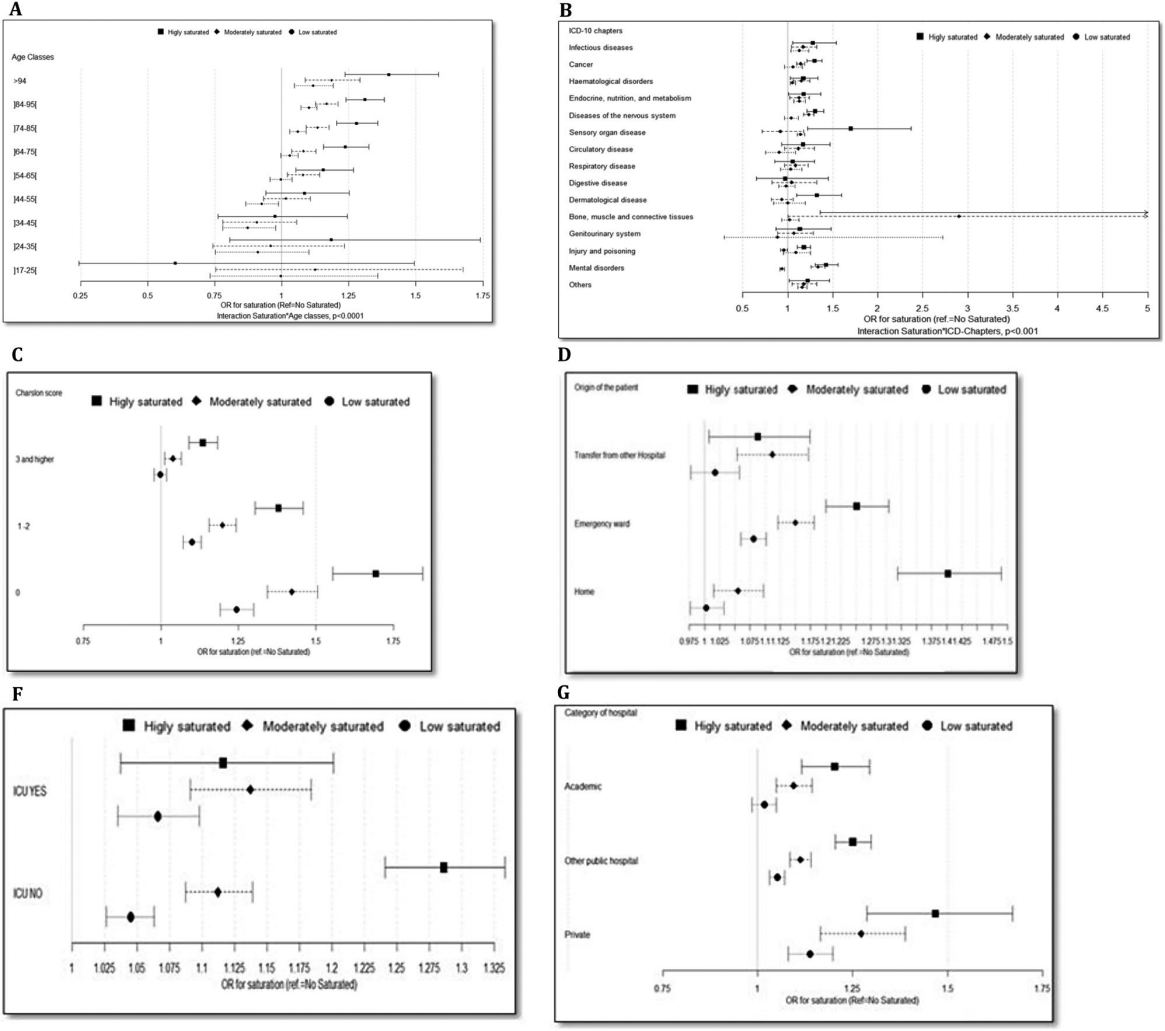
Significant interaction between hospital saturation and patients/hospital characteristics for mortality. **A** Hospital saturation*Age.Significant interaction between age and saturation: *P* < .0001. **B** Hospital saturation*ICD-10 chapters. Significant Interaction between age and ICD-10 chapters: *P* < .0001. **C** Hospital saturation*Charlson comorbidity index. Significant interaction between age and Charlson comorbidity index: *P* < .0001. **D** Hospital saturation*Origin of patients. Significant interaction between age and origin of patients: *P* < .0001. **F** Hospital saturation*Admission in intensive care unit. Significant interaction between age and ICU admission: *P* = .0009. **G** Hospital saturation*Category of hospital. Significant interaction between age and category of hospital: *P* = .0016

The results are presented in Table 2; Fig. 2, and Fig. 3. The average time from admission to death was 9.97 ± 12.5 days for deceased patients (median = 6 with an interquartile range of [2–14]).

Hospital mortality was significantly higher in hospitals with high saturation (7.02%) compared to those with moderate (5.94%), low (5.64%), and no saturation (5.30%) (Fig. 2).

The multivariate analysis confirmed these findings (Table 2): in contrast to hospital stays in non-saturated hospitals, the hospital mortality for non-COVID-19 acute medical conditions was significantly higher in low saturated hospitals (adjusted odds ratio [aOR] = 1.05, 95% CI [1.34–1.07], *P* < .001), moderate saturated hospitals (aOR = 1.12, 95% CI [1.09–1.14], *P* < .001), and highly saturated hospitals (aOR = 1.25, 95% CI [1.21–1.30], *P* < .001). Hospital mortality did not improve during the second wave compared to the first wave: aOR = 1.05 [95% CI: 1.04–1.07], *p* < .001. The VIF values ranged between 5 and 20; however, the supplementary analyses consistently yielded consistent results. The model demonstrated satisfactory performance, as evidenced by a *P* for the deviance statistic exceeding 0.99 and an AUC of 0.821 [0.820–0.822] (Supplementary Fig. 2). The findings from the three sensitivity analyses were congruent (Supplementary Table).

The proportion of deaths outside ICU was higher in highly saturated hospitals (87%) compared to non-, low- or moderate saturated hospitals (81–84%). The negative impact of hospital saturation on mortality was more pronounced (i.e., significant interaction) in patients aged 65 years and older, those with fewer comorbidities, individuals with cancer, nervous and mental diseases, patients admitted from home or through the emergency room, and those who were not admitted to the intensive care unit (Fig. 3).

### Patient case-mix

The results are presented in Table 1.

Compared to non-saturated hospitals, the casemix of highly saturated hospitals was characterized by a lower proportion of patients with cancer (13.0% vs. 9.8%), diseases of the nervous system (5.6% vs. 4.7%), circulatory diseases (22.8% vs. 19.9%) and digestive diseases (8.5% vs. 7.8%).

## Discussion

The findings of this study provide valuable insights into the impact of hospital saturation for COVID-19 care on mortality rates for non-COVID-19 acute medical conditions, highlighting the importance of improving hospital resilience, i.e., the ability of hospitals to adapt to increased demand, maintain high standards of patient care, and recover quickly from overwhelming conditions. Our study uncovers a striking “dose-effect” relationship: as hospital saturation intensifies, so does the mortality risk.

The association between hospital saturation and increased mortality rates for non-COVID-19 medical conditions is a significant concern. The negative impact of hospital saturation on mortality was particularly pronounced in patients aged 65 years and older, those with fewer comorbidities, individuals with cancer, nervous and mental diseases, patients admitted from home or through the emergency room, and those who were not admitted to the intensive care unit. It is crucial to address these specific population groups and develop targeted strategies to ensure the quality of their care and guarantee their safety. The overwhelming demand for resources and attention to COVID-19 patients may have resulted in compromised quality of care for non-COVID-19 patients as previously suggested in surgery and psychiatry [5, 9]. Several explanations have been proposed to account for these findings. The demanding work conditions resulting from staffing shortages, excessive workload, and the reallocation of resources from non-COVID-19 to COVID-19 services may have adversely affected the well-being of healthcare professionals and compromised their adherence to routine safety practices [7, 32–35]. Another explanation can be the restricted admission to the ICU, as suggested by the increased risk of death outside of the ICU in highly saturated hospitals compared to non- or low- or moderately saturated hospitals [36]. Last, delays in accessing timely and appropriate care prior to hospitalization can have significant implications for patient outcomes, including the exacerbation of medical conditions and an increased risk of adverse outcomes within the hospital setting. More specifically, we hypothesize that older individuals might have experienced more significant delays or missed care compared to younger patients due to factors such as isolation and fear [37]. Additionally, their greater vulnerability to such delays and disruptions, given their complex medical needs, might have also affected their health outcomes. Lastly, triage and access to ICU care might have disproportionately impacted this demographic [38]. Among patients with fewer comorbidities, the impact of hospital saturation on mortality might be attributed to delayed care-seeking behavior. Patients with fewer comorbidities might perceive their symptoms as less urgent compared to COVID-19 patients or those with severe comorbid conditions, potentially leading them to defer seeking medical attention. This behavior can be influenced by stay-at-home instructions from healthcare professionals or misconceptions about their overall health status. This phenomenon aligns with the concept of delayed presentation to care, as documented in the literature [39]. Likewise, patients admitted from home or through the emergency room, compared to transfers from other hospital wards, exhibited a more pronounced negative impact of hospital saturation on mortality. We hypothesize that this could be linked to the underutilization of primary healthcare services [40], leading to a potential gap between primary care and hospital care. Patients admitted from home or through the emergency room might have experienced delayed access to healthcare services, resulting in worsened outcomes upon hospitalization. Conversely, patients transferred from other hospital wards were likely already integrated into the hospital system, ensuring streamlined access to necessary care even during periods of heightened hospital activity.

While there has been an observed learning effect in surgical care during the waves of the pandemic [5], the same phenomenon has not been observed in medical acute care. The “learning phenomenon” in healthcare refers to how providers improve their efficiency and outcomes over time through gained experience and adaptation to challenges. The absence of a learning phenomenon in medical care, as suggested by the moderate increase in mortality rates between the two waves, highlights the need for further investigation and improvement in the organizational and safety aspects of medical care delivery. It is possible that the observed increase during the second wave might be due to a delayed effect of the care disruption from the previous period, linked to a decrease in screenings and appropriate management [41].

Saturated hospitals exhibited lower proportions of patients receiving care for cancer and circulatory diseases compared to non-saturated hospitals. These findings suggest that hospital saturation might have hindered access to specialized care for patients with these severe medical conditions [42, 43].

The implications of our findings underscore the need to enhance hospital resilience and patient safety in the face of future crises [44]. Fostering resilience within hospital and health care systems necessitates substantial changes and should be approached as a political priority, engaging in collective debate to shape the adopted strategy. However, France encounters challenges in prioritizing health issues politically and formulating a comprehensive long-term strategic vision [45]. It is essential to reflect on strategies that ensure adequate resource allocation, effective healthcare workforce planning, and streamlined patient flow management through enhanced coordination among health care providers. Reinforcing a territorial approach that promotes collaboration and complementarity among all care providers and patients is crucial. A public health data strategy including the data, technology, policy, and administrative actions should be reinforced to exchange critical core data efficiently and securely across healthcare and public health [46]. A thoughtful reflection on the role of digital healthcare services should be initiated [47, 48]. By collectively addressing these challenges, we can build a stronger and more resilient healthcare system based on organizational innovation [49] that effectively responds to and manages the complex and evolving healthcare crises we face.

This study has several limitations that should be acknowledged. Firstly, the calculation of saturation rates was based on declarative data regarding the number of available beds, without considering any potential addition of extra beds. However, hospitals with limited initial capacities may have less flexibility to adapt and reorganize compared to those with greater initial capacities, as it involves not only the availability of beds but also the mobilization of medical and paramedical staff. Calculating saturation rates on a weekly basis may seem arbitrary. Our aim was to strike a balance between daily and fortnightly intervals. Opting for a weekly interval offers a practical compromise, capturing short-term fluctuations and longer-term trends in hospital saturation. Importantly, this one-week timeframe aligns with the average length of patient stays, allowing for an analysis that encompasses the majority of patient stays. However, it’s important to acknowledge that the hospital saturation rate at the time of data collection may evolve in the subsequent weeks, potentially introducing some bias into our findings. We excluded patients transferred within the initial 48 h due to the tendency of highly saturated hospitals to rapidly transfer patients to other facilities (9.5% vs. 5.0% in non-saturated hospitals). This precaution was taken to prevent potential bias that could artificially lower mortality rates in such hospitals. Although our study’s scope is thus limited to a subset of the target population, our preliminary analysis including these transfers yielded results consistent with those in the manuscript. Furthermore, it is crucial to recognize that while the issue of hospital saturation and its impact during the COVID-19 period is a global concern, the findings of this study are specific to the healthcare policies and characteristics of France. Factors such as ICU capacities [36], the acceptability of government interventions including social distancing measures, isolation protocols, lockdown measures [50], prevention strategies [51, 52], quality of crisis communication [53] and safety culture [54] may vary between countries and can influence the outcomes observed. Therefore, caution should be exercised when extrapolating these results to healthcare systems in other countries. Additionally, it is important to highlight that this study did not explore the management of patients outside of the hospital setting, particularly prior to hospital admission. The impact of out-of-hospital management on hospital mortality rates should be investigated in future studies. Understanding the influence of pre-hospital care on patient outcomes is essential for a comprehensive understanding of the overall healthcare system’s response to the COVID-19 pandemic. A weakness of administrative databases is the potential miscoding of diagnoses during hospital stays, which can underestimate important patient features and disease severity, especially during saturation period. Future work should also consider investigating the impact of hospital saturation on outcomes for children and adolescents with acute medical conditions, as their distinct healthcare needs and disease profiles warrant a separate analysis. Lastly, our study exclusively includes patients who died in hospitals. However, we cannot exclude the possibility of early discharges during these periods, potentially leading to an increase in out-of-hospital deaths. This limitation must be considered when interpreting the impact and outcomes of hospital-based care in our study.

## Conclusions

Our study provides valuable insights into the consequences of hospital saturation during the COVID-19 pandemic on mortality rates for non-COVID-19 acute medical conditions. The results emphasize the importance of resilience in hospital systems and call for proactive measures to improve patient safety and strengthen healthcare infrastructure to withstand future challenges. By addressing these concerns, healthcare systems can better prepare and protect vulnerable patient populations in times of crisis.

### Electronic supplementary material

Below is the link to the electronic supplementary material.

Supplementary Material 1

## Data Availability

Anonymized participant data extracted from the nationwide hospital datawarehouse are available from the ATIH Institutional Data Access Platform forresearchers who meet the legal and ethical criteria for access to confidential data by the French national commission governing the application of data privacy laws. To obtain this dataset for an international researcher, please contact: demande_base@atih.sante.fr. All materials, including the study protocol and statistical analysis plan, are freely available.

## References

[1] Rimmelé T, Pascal L, Polazzi S, Duclos A (2021). Organizational aspects of care associated with mortality in critically ill COVID-19 patients. Intensive Care Med.

[2] Grasselli G, Zangrillo A, Zanella A, Antonelli M, Cabrini L, Castelli A (2020). Baseline characteristics and outcomes of 1591 patients infected with SARS-CoV-2 admitted to ICUs of the Lombardy Region, Italy. JAMA.

[3] Muller J, Tran Ba Loc P, Binder Foucard F, Borde A, Bruandet A, Le Bourhis-Zaimi M (2022). Major interregional differences in France of COVID-19 hospitalization and mortality from January to June 2020. Rev Epidemiol Sante Publique.

[4] Payet C, Polazzi S, Rimmelé T, Duclos A (2022). Mortality among Noncoronavirus Disease 2019 critically ill patients attributable to the pandemic in France. Crit Care Med.

[5] Duclos A, Cordier Q, Polazzi S, Colin C, Rimmelé T, Lifante J-C (2023). Excess mortality among non-COVID-19 surgical patients attributable to the exposure of French intensive and intermediate care units to the pandemic. Intensive Care Med.

[6] Davin-Casalena B, Jardin M, Guerrera H, Mabille J, Tréhard H, Lapalus D (2021). The impact of the COVID-19 epidemic on primary care in South-eastern France: implementation of a real-time monitoring system based on regional health insurance system data. Rev DÉpidémiologie Santé Publique.

[7] Dupoirier S, Dany L, Tosello B, Sorin G, Tardieu S, Dahan-Cohen S (2022). Les soignants de périnatalité face à La COVID-19: stress, qualité de vie et préoccupations perinatal caregivers coping with covid-19 : stress, quality of life and concerns. Rev DÉpidémiologie Santé Publique.

[8] Diakiese BM, Féron V (2022). [Induced abortion and COVID-19: what changed with the pandemic in 2020]. Rev Epidemiol Sante Publique.

[9] Boyer L, Fond G, Pauly V, Orléans V, Auquier P, Solmi M (2022). Impact of the COVID-19 pandemic on non-COVID-19 hospital mortality in patients with schizophrenia: a nationwide population-based cohort study. Mol Psychiatry.

[10] Mansfield KE, Mathur R, Tazare J, Henderson AD, Mulick AR, Carreira H (2021). Indirect acute effects of the COVID-19 pandemic on physical and mental health in the UK: a population-based study. Lancet Digit Health.

[11] Davies GA, Alsallakh MA, Sivakumaran S, Vasileiou E, Lyons RA, Robertson C (2021). Impact of COVID-19 lockdown on emergency asthma admissions and deaths: national interrupted time series analyses for Scotland and Wales. Thorax.

[12] Ball S, Banerjee A, Berry C, Boyle JR, Bray B, Bradlow W (2020). Monitoring indirect impact of COVID-19 pandemic on services for cardiovascular diseases in the UK. Heart.

[13] Kubica J, Ostrowska M, Stolarek W, Kasprzak M, Grzelakowska K, Kryś J (2022). Impact of COVID-19 pandemic on acute heart failure admissions and mortality: a multicentre study (COV‐HF‐SIRIO 6 study). ESC Heart Fail.

[14] Valabhji J, Barron E, Gorton T, Bakhai C, Kar P, Young B (2022). Associations between reductions in routine care delivery and non-COVID-19-related mortality in people with diabetes in England during the COVID-19 pandemic: a population-based parallel cohort study. Lancet Diabetes Endocrinol.

[15] Wichmann B, Moreira Wichmann R (2023). Big data evidence of the impact of COVID-19 hospitalizations on mortality rates of non-COVID-19 critically ill patients. Sci Rep.

[16] McLarty J, Litton E, Beane A, Aryal D, Bailey M, Bendel S, et al. Non-COVID-19 intensive care admissions during the pandemic: a multinational registry-based study. Thorax. 2023. thorax-2022-219592. 10.1136/thorax-2022-219592 37225417

[17] Mazzilli S, Scardina G, Collini F, Forni S, Gianolio G, Bisceglia L (2023). Hospital admission and mortality rates for non-covid diseases among residents of the long-term care facilities before and during the pandemic: a cohort study in two Italian regions. J Public Health.

[18] Chaurasia A, Gupta D, Shweta K, Srivastava Y (2023). Impact of COVID-19 pandemic on Non-COVID-19 maternal mortalities in a Tertiary Health Care Center of North India. J Obstet Gynecol India.

[19] https://. drees.solidarites-sante.gouv.fr/sites/default/files/2021-07/ES2021.pdf.

[20] Gutton J, Lin F, Billuart O, Lajonchère J-P, Crubilié C, Sauvage C (2022). L’intelligence Artificielle Au service des départements d’information médicale: construction et évaluation d’un outil d’aide à La décision pour cibler et prioriser les séjours à contrôler et fiabiliser les recettes hospitalières générées par La Tarification à l’activité. Rev DÉpidémiologie Santé Publique.

[21] Boudemaghe T, Belhadj I. Int J Epidemiol. 2017;46:392–d392. Data Resource Profile: The French National Uniform Hospital Discharge Data Set Database (PMSI). 10.1093/ije/dyw359 28168290

[22] Benchimol EI, Smeeth L, Guttmann A, Harron K, Moher D, Petersen I (2015). The REporting of studies conducted using Observational routinely-collected health data (RECORD) Statement. PLOS Med.

[23] Kadri SS, Gundrum J, Warner S, Cao Z, Babiker A, Klompas M (2020). Uptake and accuracy of the diagnosis code for COVID-19 among US hospitalizations. JAMA.

[24] Sudre C, Duplan H, Bukasakakamba J, Nacher M, Peyre-Costa P, Sabbah N (2021). Diabetes Care in French Guiana: the gap between National guidelines and reality. Front Endocrinol.

[25] https://data.drees.solidarites-sante.gouv.fr/explore/dataset/707_bases-administratives-sae/information/?sort=-annee.

[26] Quan H, Li B, Couris CM, Fushimi K, Graham P, Hider P (2011). Updating and validating the Charlson Comorbidity Index and score for Risk Adjustment in Hospital discharge abstracts using data from 6 countries. Am J Epidemiol.

[27] Lakbar I, Leone M, Pauly V, Orleans V, Srougbo KJ, Diao S (2023). Association of severe mental illness and septic shock case fatality rate in patients admitted to the intensive care unit: a national population-based cohort study. PLOS Med.

[28] Fabre C, Pauly V, Baumstarck K, Etchecopar-Etchart D, Orleans V, Llorca P-M (2021). Pregnancy, delivery and neonatal complications in women with schizophrenia: a national population-based cohort study. Lancet Reg Health - Eur.

[29] Fond G, Salas S, Pauly V, Baumstarck K, Bernard C, Orleans V (2019). End-of-life care among patients with schizophrenia and cancer: a population-based cohort study from the French national hospital database. Lancet Public Health.

[30] Boyer L, Fond G, Gauci M-O, Boussat B (2023). Regulation of medical research in France: striking the balance between requirements and complexity. Rev DÉpidémiologie Santé Publique.

[31] https://www.health-data-hub.fr/projets/etude-en-vie-reelle-de-limpact-de-la-pandemie-covid-19-sur-lacces-et-la-qualite-de-la-prise.

[32] Rollin L, Gehanno J-F, Leroyer A (2022). Occupational stressors in healthcare workers in France. Rev DÉpidémiologie Santé Publique.

[33] Papazian L, Hraiech S, Loundou A, Herridge MS, Boyer L (2023). High-level burnout in physicians and nurses working in adult ICUs: a systematic review and meta-analysis. Intensive Care Med.

[34] Bruyneel A, Smith P, Tack J, Pirson M (2021). Prevalence of burnout risk and factors associated with burnout risk among ICU nurses during the COVID-19 outbreak in French speaking Belgium. Intensive Crit Care Nurs.

[35] Erraoui M, Lahlou L, Fares S, Abdelnaby A, Nainia K, Ajdi F (2022). The impact of COVID-19 on the quality of life of southern Moroccan doctors: a gender-based approach. Rev DÉpidémiologie Santé Publique.

[36] Annane D, Federici L, Chagnon J-L, Diehl JL, Dreyfuss D, Guiot P (2021). Intensive care units, the Achilles heel of France in the COVID-19 battle. Lancet Reg Health Eur.

[37] Soares P, Leite A, Esteves S, Gama A, Laires PA, Moniz M (2021). Factors Associated with the patient’s decision to avoid Healthcare during the COVID-19 pandemic. Int J Environ Res Public Health.

[38] Akinosoglou K, Schinas G, Almyroudi MP, Gogos C, Dimopoulos G (2023). The impact of age on intensive care. Ageing Res Rev.

[39] Nab M, van Vehmendahl R, Somers I, Schoon Y, Hesselink G (2021). Delayed emergency healthcare seeking behaviour by Dutch emergency department visitors during the first COVID-19 wave: a mixed methods retrospective observational study. BMC Emerg Med.

[40] Tuppin P, Lesuffleur T, Constantinou P, Atramont A, Coatsaliou C, Ferrat E (2022). Underuse of primary healthcare in France during the COVID-19 epidemic in 2020 according to individual characteristics: a national observational study. BMC Prim Care.

[41] Blanpain N. 53 800 décès de plus qu’attendus en 2022: une surmortalité plus élevée qu’en 2020 et 2021. Insee Première. 2023.

[42] Walker MJ, Wang J, Mazuryk J, Skinner S-M, Meggetto O, Ashu E (2022). Delivery of Cancer Care in Ontario, Canada, during the First Year of the COVID-19 pandemic. JAMA Netw Open.

[43] Driggin E, Madhavan MV, Bikdeli B, Chuich T, Laracy J, Biondi-Zoccai G (2020). Cardiovascular considerations for patients, Health Care Workers, and Health systems during the COVID-19 pandemic. J Am Coll Cardiol.

[44] Fallah-Aliabadi S, Ostadtaghizadeh A, Ardalan A, Fatemi F, Khazai B, Mirjalili MR (2020). Towards developing a model for the evaluation of hospital disaster resilience: a systematic review. BMC Health Serv Res.

[45] Cremieux F. Health, a poorly identified political object. Esprit. 2023;:89–93.

[46] Office of Public Health Data. Surveillance, and Technology (OPHDST). Public Health Data Strategy: Public Health Data Goals and 2-Year Milestones. 2023.

[47] Baudier P, Kondrateva G, Ammi C, Chang V, Schiavone F (2023). Digital transformation of healthcare during the COVID-19 pandemic: patients’ teleconsultation acceptance and trusting beliefs. Technovation.

[48] Ouédraogo S, Accrombessi M, Ouattara A, Massougbodji A, Dabira ED, Sarigda M (2022). Impact of mobile phone intervention on intermittent preventive treatment of malaria during pregnancy in Burkina Faso: a pragmatic randomized trial. Rev DÉpidémiologie Santé Publique.

[49] Stevens N, Cambon L, Bataillon R, Robin S, Alla F (2022). Décrire L’innovation organisationnelle en santé publique pour favoriser sa dissémination; guide DINOSP (description des innovations organisationnelles en santé publique). Rev DÉpidémiologie Santé Publique.

[50] Diallo AI, Faye A, Tine JAD, Ba MF, Gaye I, Bonnet E (2022). Factors associated with the acceptability of government measures to address COVID-19 in Senegal. Rev DÉpidémiologie Santé Publique.

[51] Montagni I, Ouazzani-Touhami K, Pouymayou A, Pereira E, Texier N, Schück S (2022). Who is hesitant about Covid-19 vaccines? The profiling of participants in a French online cohort. Rev DÉpidémiologie Santé Publique.

[52] Melchior M, Desgrées du Loû A, Gosselin A, Datta GD, Carabali M, Merckx J (2021). À quand une prise en compte des disparités ethnoraciales vis-à-vis de l’infection à COVID-19 en France ?. Rev DÉpidémiologie Santé Publique.

[53] Ben Abdelaziz A, El Haddad N, Hannachi H, Nouira S, Melki S, Chebil D (2021). Qualité Des supports de communication de crise lors de la pandémie de la COVID-19 Au Grand Maghreb. Rev DÉpidémiologie Santé Publique.

[54] Shih C, Buchet-Poyau K, Keriel-Gascou M, Quenon J-L, Michel P, Touzet S (2022). Culture de sécurité des professionnels de santé en soins primaires: adaptation en langue française du questionnaire MOSPSC (« Medical Office Survey on Patient Safety Culture »). Rev DÉpidémiologie Santé Publique.

