# Supplementary material for: The impact of hospital saturation on non-COVID-19 hospital mortality during the pandemic in France: a national population-based cohort study
Source: BMC Public Health. 2024 Jul 5;24:1798. doi: 10.1186/s12889-024-19282-3 (PMC11227237; doi:10.1186/s12889-024-19282-3)
Supplement: Supplementary file 1 — Supplementary Material 1 [file 12889_2024_19282_MOESM1_ESM.docx]

**Supplementary Figure 1.** COVID-19 saturation rates.

**
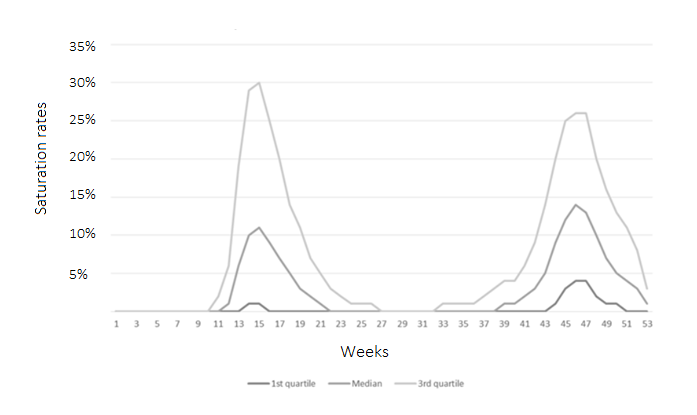
**

**Supplementary Figure 2.** Receiver Operating Characteristic (ROC) Curve and Area Under the Curve (AUC) for Logistic Model.

**
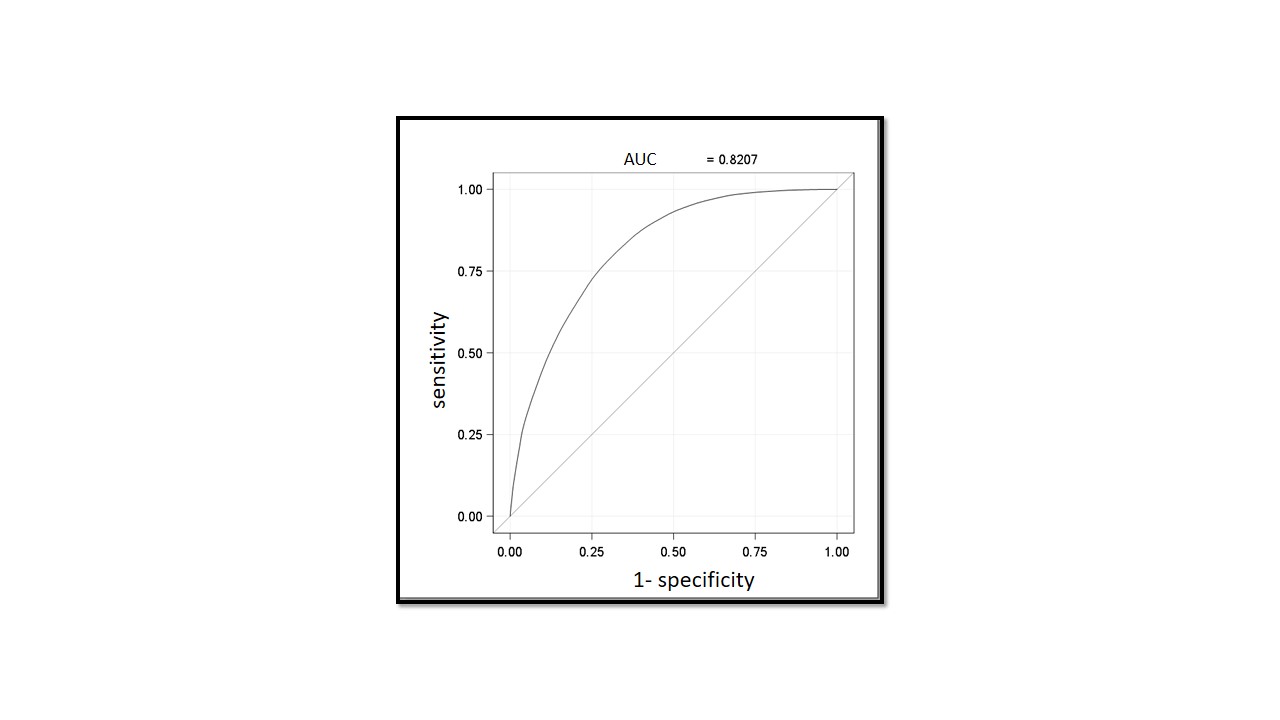
**

**Supplementary Tables.** Sensitivity analyses.

|  | [5-15%[ (low) | [15-30%[ (moderate) | >30% (high) |
| --- | --- | --- | --- |
| Multivariate analysis was conducted, incorporating all covariates and replacing the Charlson Comorbidity Index with 17 indicator variables representing individual comorbidities (reference <5% (no)) | 1.050 [1.034-1.067] | 1.121 [1.098-1.145] | 1.255 [1.214-1.297] |

|  | Univariate OR  [95% CI] | *P* | Multivariate OR*  [95% CI] | *P* |
| --- | --- | --- | --- | --- |
| Multivariate analysis was conducted including saturation rate as continuous numeric variable | 1,824 [1,721-1,933] | <.0001 | 1,677 [1,577-1,783] | p<.0001 |

|  | [5-15%[ (low) | [15-30%[ (moderate) | >30% (high) |
| --- | --- | --- | --- |
| Multivariate analysis was conducted using Cox regression model with shared frailty model (reference <5% (no)) | 1.051  [1.036-1.066] | 1.136 [1.114-1.158] | 1.267 [1.230-1.304] |
